# Adipose-derived stromal cells for osteoarticular repair: trophic function versus stem cell activity

**DOI:** 10.1017/erm.2014.9

**Published:** 2014

**Authors:** M. Ruetze, W. Richter

**Affiliations:** Research Centre for Experimental Orthopaedics, Heidelberg University Hospital, Schlierbacher Landstrasse 200a, D-69118 Heidelberg, Germany

## Abstract

The identification of multipotent adipose-derived stromal cells (ASC) has raised hope that tissue regeneration approaches established with bone-marrow-derived stromal cells (BMSC) can be reproduced with a cell-type that is far more accessible in large quantities. Recent detailed comparisons, however, revealed subtle functional differences between ASC and BMSC, stressing the concept of a common mesenchymal progenitor existing in a perivascular niche across all tissues. Focussing on bone and cartilage repair, this review summarises recent in vitro and in vivo studies aiming towards tissue regeneration with ASC. Advantages of good accessibility, high yield and superior growth properties are counterbalanced by an inferiority of ASC to form ectopic bone and stimulate long-bone healing along with their less pronounced osteogenic and angiogenic gene expression signature. Hence, particular emphasis is placed on establishing whether stem cell activity of ASC is so far proven and relevant for successful osteochondral regeneration, or whether trophic activity may largely determine therapeutic outcome.

## Introduction

Established strategies for cartilage and bone repair, such as autologous chondrocyte transplantation (ACT) (Ref. 1) and bone grafting (Ref. 2), have reached broad clinical application and yield satisfactory results due to continuous improvement. These therapies, however, require the excision of healthy tissue from a nonlesioned site, necessarily incorporating the disadvantages of additional medical procedures, donor site morbidity and further rehabilitative burden on the patient (Ref. 3). Repair strategies that are based on autologous bone-marrow-derived stromal cells (BMSC) do not circumvent these problems, but harvesting bone marrow from the iliac crest is generally judged as less invasive (Ref. 4). The discovery that multipotent stromal cells can be isolated from lipoaspirates (Ref. 5) and that the number of adherent cells in an equal volume of adipose tissue exceeds the content of bone marrow aspirate by about 300-fold (Refs 6, 7, 8) challenged the assumption that bone marrow would be the most appropriate source for cell-based therapies of skeletal injuries and diseases.

In order to verify whether adipose-derived stromal cells (ASC) represent an easily accessible cell type that may substitute for BMSC completely in cell-based approaches for osteochondral regeneration, they were characterised in terms of in vitro performance (Refs 9, 10), in vivo localisation (Refs 11, 12) and their ability to differentiate into various mesenchymal cell types (Refs 13, 14, 15, 16). This review summarises current knowledge of ASC and BMSC plasticity and in vivo function, describing similarities and differences between both cell types that have been determined upon expansion. Furthermore, an overview is provided on osteoarticular regenerative approaches that have thus far been conducted using ASC. In summary, data on ASC-based osteoarticular repair strategies indicate that ASC do not possess intrinsic osteochondral potential, such as BMSC, but require reprogramming for in vivo development towards the osteochondral lineage. These observations stress the concept of equivalent mesenchymal progenitors in bone marrow and adipose tissue (Ref. 8). In view of a long list of successful experimental intervention studies in distinct models, trophic functions of ASC may be more relevant than stem cell potential in mediating osteoarticular repair.

## Stemness of BMSC and ASC

### Criteria for stem cell definition

Thus far absent from the literature is a comprehensive, general convention that defines intrinsic properties for stem cells of any given tissue (Ref. 17). From a functional point of view, a well-accepted interpretation would be that a single stem cell possesses the capacity to build up a physiological, multicellular tissue that is capable of autonomous regeneration in vivo. Specific cellular functions such as asymmetric cell division, prolonged self-renewal and differentiation capacities are needed to fulfil this requirement. Most importantly, in vitro detection of these properties in a particular cell type alone, however, does not necessarily prove stemness. It is self-explanatory that a stem cell only deserves this designation if the observed fundamental capacities represent intrinsic features of the native cell in vivo, rather than being achieved by artificial treatments or molecular reprogramming. These stringent criteria for stem cell definition (Ref. 18) are met by haematopoietic stem cells (HSC), which reconstitute bone marrow when clonally derived HSC are transplanted into lethally irradiated mice (Ref. 19). In the context of osteoarticular repair, BMSC are so far the only entity representing skeletal stem cells, according to this stringent definition. Sacchetti et al. established that clonal BMSC populations are self-renewing and can form an ectopic bone organ after subcutaneous transplantation into immunocompromised mice (Ref. 20). This result was further refined by Chan et al. by demonstrating the formation of multicellular bone tissue at other ectopic sites and by unravelling routes of differentiation from a discrete progenitor subpopulation to cell types that either contribute to bone, cartilage or haematopoiesis-supportive stroma within the new bone organ (Ref. 21). Besides BMSC and HSC, a plethora of other cell types is commonly designated as stem cells, although evidence for clonal in vivo organ formation without pre-induction is missing. This circumstance also holds true for ASC that, nevertheless, are often referred to as *adipose-derived mesenchymal stem cells*, despite the fact that the capacity of a single ASC to build up a functional mesenchymal tissue has not been shown to date. Thus, the idea of universal mesenchymal stem cells in a perivascular niche (Ref. 22) with an intrinsic capacity to build up and maintain multiple mesenchymal tissues (Ref. 8) by a common mesengenic in vivo process is still an unconfirmed hypothesis. Conclusively, BMSC are the only perivascular cells with proven skeletal stem cell characteristics.

### Lack of evidence for stem cell characteristics of ASC

Current data do not exclude that ASC may possess stem cell characteristics, according to the stringent criteria outlined above, and results are encouraging that future experiments may support adipose tissue-specific stem cell properties. In vitro characterisation of isolated ASC demonstrated extensive proliferative potential (Ref. 5). The application of standard in vitro differentiation protocols to ASC reflected possession of osteogenic, adipogenic and chondrogenic differentiation capacity (Ref. 5), although the physiological relevance of these assays has been questioned (Ref. 18). Notably, clonal analyses revealed that >2% of cells within expanded ASC cultures exhibit tri-lineage potential in vitro, indicating that typical isolation protocols lead only to a small fraction of ASC with in vitro multilineage potential after artificial treatment (Ref. 10).

Like the most mesenchymal cells (Ref. 23), ASC express a cell surface marker profile that is comparable to BMSC (Refs 9, 24), fulfilling all requirements that have been suggested as the minimal criteria for defining multipotent mesenchymal stromal cells (Ref. 25). Although most mesenchymal cells also meet these standards (Refs 23, 26), a functional equivalence of ASC and BMSC was construed from this classification and subsequent studies focussed on the question of whether ASC exhibit analogous cartilage and bone regeneration capacities. For this purpose, ASC were directly tested for their application to osteochondral in vivo repair approaches; however, the question whether ASC are skeletal stem cells functionally equivalent to BMSC attracted little interest.

From a retrospective point of view, it seems inconsistent that ASC were first assessed for their cartilage and bone regeneration potential before their capacity to build up and maintain a physiological adipose tissue environment was investigated. Although in vitro engineered adipose tissue would have a promising potential for surgical soft tissue reconstruction (Ref. 27), strategies using ASC for that purpose are instead clearly outnumbered by approaches that use (pre-)adipocytes (Refs 28, 29, 30, 31) or even BMSC (Refs 32, 33, 34). Beside studies that address in vitro engineering of adipose tissue from ASC (Ref. 35), ASC were seeded in fibrin (Ref. 36), alginate (Ref. 37) or collagen scaffolds (Ref. 38) and subjected to an adipogenic pre-induction protocol prior to subcutaneous implantation. As expected after pre-induction, in vivo adipose tissue formation was reported in these studies. Evidence that transplanted clonal ASC can generate adipose tissue in vivo without such a pre-induction is still missing, but such a demonstration would not only be encouraging for their use in adipose tissue engineering but would also further clarify if ASC may indeed represent tissue-specific stem cells distinct from BMSC.

## In vitro characteristics of expanded ASC and BMSC

### Similar morphological features but different growth behaviour

A thorough review of the literature on in vitro performance of culture-expanded ASC and BMSC reveals strong similarities between stromal cells of both sources, factually overweighing the differences. For instance, no morphological differences were reported to date, and the same spindle-shaped phenotype was frequently described (Refs 39, 40, 41). Upon isolation, adherent human and mouse ASC seem to exhibit a higher proliferation rate (Refs 41, 42, 43, 44, 45), but equal growth behaviour compared to BMSC has also been reported (Refs 40, 46). In an extended comparison of growth kinetics, Dmitrieva et al. included the fact that the amount of colony-forming cells in the adipose-derived stromal vascular fraction (SVF) exceeds that of the bone marrow nucleated fraction (BM-NC) by at least two orders of magnitude (Refs 42, 43, 47). This leads to the result that ASC underwent significantly less population doublings up to the first passage, even if, as usual, more primary BM-NC were initially plated. It could therefore be speculated that signs of senescence only occur later in ASC (Ref. 43) because BMSC underwent more cell divisions in the same passage number. However, Dmitrieva et al. showed that ASC indeed possess extended proliferative potential, since more population doublings were observed before cells acquired a senescent phenotype (Ref. 42).

### Dissimilar expression of cell surface markers CD106, CD146 and CD34

Extensive analyses have been conducted to map differences between cultured ASC and BMSC with regard to surface marker expression, leading to a reliable picture in which markers distinguish expanded stromal cells of both sources. Again, it is striking that, despite the application of large panels of antibodies (Refs 48, 49, 50), just a few CD markers are differently expressed between ASC and BMSC. Multiple reports describe higher expression of CD106 in BMSC (Refs 40, 43, 47, 48, 50, 51, 52, 53), whereas there exist some studies in which no difference was detected (Refs 49, 54). Analogous data exist for CD146, a cell surface marker of pericytes (Ref. 55) that has been used to enrich for multipotent cells (Refs 56, 57) and to identify the localisation of multipotent stromal cells in various tissues (Refs 12, 20, 22, 58). Adherent stromal cells from bone marrow and adipose tissue both contain a CD146-positive population, but this fraction is about twofold larger in early passage BMSC (Refs 42, 50, 53, 59).

By means of a quality control check that is commonly performed at the beginning of studies, ASC were frequently analysed for CD34-negativity, since the absence of this marker is a prerequisite to meet the minimal criteria for multipotent mesenchymal stromal cells (Refs 25, 47). However, several reports that dealt with a comparison of ASC and BMSC described that adherent ASC included a substantial CD34-positive fraction, whereas BMSC that were analysed in parallel were completely CD34-negative (Refs 48, 50, 52, 60, 61). In these studies, the selection of adherent cells from the adipose tissue-derived SVF at first led to a twofold enrichment of CD34-positive cells (Ref. 61), followed by a gradual decrease in subsequent passages (Ref. 24). Nevertheless, a considerable number of CD34-positive cells was still detected in passage 4 (Refs 48, 52), and Yoshimura et al. even described that after 20 weeks of cultivation, almost 20% of the ASC population was still CD34-positive. These inconsistent data on CD34 expression in ASC cultures may simply reflect that, depending on tissue source and isolation protocol, CD34-positive endothelial cells were occasionally included in primary isolates and gradually disappeared with culture time, due to unfavoured growth conditions. It remains to be determined, however, if this hypothesis or the choice of an antibody of the appropriate subclass (Ref. 47) accounts for the conflicting results. In any case, the general absence of CD34 in BMSC cultures represents another noticeable difference compared to ASC preparations.

Compared to the CD markers that were discussed above, considerably less experimental data indicate differential expression of CD10 (Ref. 47), CD133 (Ref. 40), CD54 (Ref. 48), HLA-ABC (Ref. 53) and CD49d/f (Refs 48, 50) between BMSC and ASC. In summary, the most substantiated differences regarding cell surface proteins are lower expression levels of CD106 (VCAM-1) and CD146 (MCAM) in ASC versus BMSC, both of which point to a less angiogenic signature of ASC that reoccurs in their gene expression profile as discussed below.

### Reduced osteogenic gene expression signature in ASC

In terms of global gene and protein expression profiling, comparisons using cDNA microarrays and two-dimensional electrophoresis revealed high consistencies between multiclonal ASC and BMSC cultures (Refs 52, 62, 63). Nevertheless, hierarchical clustering of protein and gene expression data allowed for a separation of ASC and BMSC specimen, possibly due to the observation that Wnt-signalling-associated genes are more abundant in BMSC (Ref. 52). Increased expression levels of genes that are associated with osteogenesis were detected in BMSC (Refs 62, 64), arguing for a higher degree of osteochondral commitment (Ref. 59). A similar finding was made by mRNA representational difference analysis, where higher ITM2A expression in ASC could be attributed to a lower chondrogenic potential (Ref. 65). An enrichment of genes that are involved in angiogenic signalling pathways was reported for BMSC (Ref. 66) and confirmed by higher expression of angiogenic markers, such as angiopoietin and vascular endothelial growth factor (VEGF) in comparison to ASC (Ref. 59). On the other hand, ASC were shown to exhibit a more adipogenic gene expression pattern (Ref. 64), illustrated by higher expression levels of adiponectin and visfatin (Ref. 59). In line with the higher proliferation rate of ASC, genes involved in mitosis and DNA replication are also up-regulated compared to BMSC (Ref. 67).

### Reduced performance of ASC in osteochondral in vitro differentiation assays

In line with indications of an intrinsic osteogenic potential of BMSC, exposure to common osteogenic differentiation media induced more mineralisation (Refs 40, 50, 68, 69), higher alkaline phosphatase activity (Refs 40, 44, 68) and stronger gene expression of osteogenic markers, such as runx2, osteocalcin, osterix, alkaline phosphatase and collagen-1 (Refs 40, 44, 52), compared to ASC. In turn, and corresponding to their physiological origin, ASC seem to exhibit a higher affinity to adipogenic differentiation, since inclusion of lipid droplets (Refs 44, 50, 53) and expression of the adipogenic marker gene peroxisome proliferator-activated receptor (PPARγ) (Refs 44, 53) were more intense than in BMSC upon induction. However, similar adipogenic in vitro differentiation capacities of adipose and bone marrow-derived cells were also reported (Refs 52, 69, 70), but no study described a higher adipogenic potential for BMSC. In line with better in vitro osteogenesis, BMSC also showed better performance in common chondrogenesis assays. In vitro differentiation of BMSC in 3D-pellet culture under treatment with TGF-β resulted in more intense collagen-II staining (Refs 39, 46, 62, 68, 69, 70), proteoglycan deposition (Refs 39, 46, 53, 62, 68, 70) and gene expression of Sox-9 (Ref. 53), compared to ASC. Interestingly, the inferior chondrogenic capacity of ASC can be augmented to BMSC levels when BMP-6 is added to the differentiation medium (Refs 62, 71), an observation with obvious relevance for future in vivo applications of ASC.

## Comparison of trophic activity

One main path to tissue reconstruction by cell-based therapeutic strategies involves stem cell activity to establish and build new tissue by proliferating and differentiating cells, which are progeny of the implanted cells. A second way to regeneration is the stimulation of endogenous healing capacity by trophic activity of implanted cells, which attract host progenitor cells and organise repair by local and invading cells. Implanted cells may even disappear after this task has been successfully fulfilled. In this second scenario, even transient stem cell activity or differentiation capacity within target tissues may be dispensable as long as trophic activity is high.

Similar to the established trophic role of BMSC (Refs 72, 73), cultured ASC were shown to secrete a wide range of proteins (Ref. 74) into conditioned media that predominantly exert anti-apoptotic (Refs 75, 76, 77), immunomodulatory (Refs 78, 79, 80) and angiogenic (Refs 77, 81, 82) effects on co-cultured cell types. Extracellular matrix components and secreted enzymes comprised the largest fraction of the secretome, according to mass spectrometry (Refs 83, 84, 85), but these molecules are unlikely to be heavily involved in the observed paracrine signalling. These effects are instead mediated by secreted cytokines that typically appear in nano- or picomolar concentrations. Corresponding ELISA and multiplex approaches primarily identified VEGF (Refs 86, 87, 88), hepatocyte growth factor (HGF) (Refs 82, 89) and insulin-like growth factor-1 (IGF-1) (Refs 77, 90) as factors that are responsible for the described intercellular communication. According to this repertoire, both ASC and BMSC can be expected to display trophic functions (Refs 74, 91), delineating their potential to stimulate bone and cartilage regeneration solely by trophic mechanisms.

## In vivo comparison of ASC and BMSC

The extent of the described in vitro differences between ASC and BMSC gives the impression that cells of both sources may fundamentally differ from each other. This point of view must be carefully considered, since in vitro variances may stem from dissimilar donor tissue processing, cell isolation protocols, cell yield and culture methods. In the context of osteochondral regeneration, the proof of in vivo exchangeability of ASC and BMSC is far more important, and aspects of in vivo stem cell activity like trophic activity should be considered, as long as precise healing mechanisms are unclear for the diverse application settings.

### Untreated ASC do not form ectopic bone

Ectopic bone formation is a standard activity of human BMSC on calcium phosphate ceramics such as β-tricalcium phosphate (β-TCP) and hydroxyapatite (HA)/TCP in immunodeficient mice (Refs 20, 92, 93, 94, 95, 96, 97) with no osteogenic pre-induction protocols required, in line with skeletal stem cell activity of BMSC. Ectopic transplantation of ASC reliably led to de novo generation of bone when cells were subjected to osteogenic pre-induction before implantation (Refs 45, 98, 99, 100, 101). Overexpression of BMP-2/RUNX2 (Ref. 102) or BMP-7 (Ref. 103) in ASC allowed the omission of the pre-differentiation step.

Whether ASC possess the same intrinsic ability to form ectopic bone without any of these pre-treatments in standard assays, and to what extent they build up new bone themselves, largely remained elusive until the issue was recently addressed by Brocher et al. In this first standardised comparison of BMSC and ASC on multiple donors, ASC generated no ectopic bone on osteoconductive scaffolds, while samples from all BMSC donors formed ossicles under identical conditions (Ref. 59). The additional value of this ASC study was the reliable identification of donor as well as host cells within the ectopic setting via a recently established in situ hybridisation technique, specifically marking highly repetitive DNA sequences of human as well as mouse origin (Ref. 104). This demonstrated that ectopic bone was formed by ASC when they were subjected to chondrogenic pre-differentiation before transplantation. Although, in noninduced samples, ASC survived at the ectopic site for more than 8 weeks and they did not form bone, as seen in previous studies (Refs 45, 99, 100, 102, 105) (Table 1). Only Zannettino et al. have provided convincing data of ectopic bone formation after ASC implantation on HA/TCP in NOD/SCID mice, when CD146-pre-sorted cells were transplanted; however, the origin of bone from donor or host remained unclear (Ref. 12).

**Table 1.** Summary of ectopic bone formation studies with ASC

Pre-induction	ASC donor species	Acceptor species	Scaffold	Author
Yes	Human	Mouse	β-TCP	Brocher et al. (Ref. 59)
Yes	Human	Mouse	Healos^©^	Niemeyer et al. (Ref. 149)
Yes	Human	Mouse	Collagen- HA/TCP	Hicok et al. (Ref. 150)
Yes	Human	Mouse	β-TCP	Hattori et al. (Ref. 99)
Yes	Human	Mouse	ACHMS	Hattori et al. (Ref. 100)
Yes	Pig	Rat	BA	Schubert et al. (Ref. 45)
Yes	Rabbit^a^	Rabbit	COL/PLGA–β-TCP	Hao et al. (Ref. 98)
Yes	Rat	Mouse	NanoBCP	Lin et al. (Ref. 101)
Yes^b^	Human	Mouse	PLGA	Lee et al. (Ref. 102)
Yes^b^	Rat	Rat	Collagen	Yang et al. (Ref. 103)
No	Human	Mouse	HA/TCP	Zannettino et al. (Ref. 12)
No	Human	Mouse	ENGIpore^©^	Scherberich et al. (Ref. 105)
No	Human	Rat	DBM	Supronowicz et al. (Ref. 151)
No	Dog^a^	Dog	BCP	Yao et al. (Ref. 152)

^a^Autologous setting.

^b^Transgene expression.

Abbreviations: ACHMS, atelocollagen honeycomb-shaped scaffold with membrane seal; BA, bone allograft; BCP, biphasic calcium phosphate; COL, collagen; DBM, demineralised bone matrix; HA, hydroxyapatite; PLGA, polylactic acid/polyglycolic acid co-polymer; β-TCP, β-tricalcium phosphate.

All in all, beyond their reduced performance in osteochondral in vitro differentiation assays, ASC showed no intrinsic osteochondral in vivo differentiation potential and, thus, seem to possess no skeletal stem cell properties as seen with BMSC, providing a strong argument for fundamental functional differences regarding their use for in vivo osteochondral repair. Since nonclonal cells are widely used for tissue regeneration, the benefit of enhanced availability of ASC, therefore, appears currently to be balanced by an enhanced need for inductive conditions via timely and intensive in vitro culture efforts, if their physical contribution to the new skeletal tissue is desired.

### ASC and BMSC require pre-differentiation for ectopic cartilage formation

The most convincing demonstration of spontaneous chondrogenic in vivo potential of ASC and BMSC derives from observations of ectopic cartilage deposits in assays, in which articular chondrocytes form cartilaginous nodules at ectopic sites in the absence of chondrogenic inducers. Importantly, such activity has so far neither been demonstrated in human BMSC nor ASC, and an apparent necessary aspect of common strategies for successful ectopic cartilage formation includes chondrogenic pre-differentiation. Several studies have been performed in which human ASC were either used without scaffolds (Refs 62, 106) or seeded on hydrogels (Refs 106, 107, 108) or glycolic acid/lactic acid copolymer (PLGA) (Refs 109, 110, 111). In addition, different strategies were used for chondrogenic pre-differentiation of ASC (Table 2). All included TGF-β treatment either during 3D pellet culture (Refs 62, 107, 108), in vitro cultivation of ASC in the scaffold (Refs 110, 111) or by adenoviral TGF-β overexpression (Ref. 109) before subcutaneous implantation into immunocompromised mice. Ectopic cartilage composed of implanted cells was observed in all cases, but chondrocyte hypertrophy and matrix calcification were unwanted side effects reminiscent of growth plate chondrocytes (Refs 62, 112). In conclusion, analogous studies without chondrogenic (pre-) induction have remained unsuccessful, with neither BMSC nor ASC displaying intrinsic chondrogenic potential nor trophic activity leading to generation of ectopic cartilage of donor or host origin, compared to chondroprogenitors from cartilage which do display such activity (Ref. 113).

**Table 2.** Summary of ectopic cartilage formation studies with ASC

Pre-induction	ASC donor species	Acceptor species	Scaffold	Author
Yes	Human	Mouse	PLGA	Mehlhorn et al. (Ref. 111)
Yes	Human	Mouse	fibrin	Yoon et al. (Ref. 108)
Yes	Human	Mouse	PLCL/fibrin	Jung et al. (Ref. 110)
Yes^a^	Human	Mouse	PLGA/alginate	Jin et al. (Ref. 109)

^a^Transgene expression.

Abbreviations: PLCL, polylactic acid/polycaprolactone co-polymer; PLGA, polylactic acid/polyglycolic acid co-polymer.

### Missing evidence for physical ASC contribution to the repair of damaged cartilage

The most direct and least invasive approach to use ASC for the treatment of cartilage defects is by intra-articular injection of cells. Studies that started with an induction of osteoarthritis (OA) by anterior cruciate ligament transection (ACLT) or collagenase treatment, followed by intra-articular injection of autologous ASC, have been conducted in mouse (Ref. 114) and rabbit (Refs 115, 116) (Table 3). Different histological evaluations and OA scoring scales were used to measure OA progression, but in all cases, positive effects of ASC compared to the injection of cell-free solvent were reported. Labelled ASC were detectable in the synovial membrane and medial meniscus 20 days after injection (Ref. 115) and at the synovial lining and cruciate ligaments up to 5 days after injection (Ref. 114). Human ASC injected into unimpaired mouse knee joints showed long-term persistence in joint tissue in 60% of all mice up to 186 days after injection, but a substantial fraction of ASC seemed to have migrated to the bone marrow, adipose tissue and muscle. Thus, while a certain degree of persistence of injected cells can therefore be assumed, evidence for in vivo differentiation of donor ASC or long-term integration into articular cartilage tissue is missing, and contributions by trophic activity cannot be judged.

**Table 3.** Summary of orthotopic cartilage formation studies with ASC

Defect	ASC donor species	Acceptor species	Scaffold	Pre-induction	Long-term engraftment	Author
Femur	Pig^a^	Pig	PGA/PLA	Yes	n.d.	Cui et al. (Ref. 119)
Femur	Rabbit^a^	Rabbit	PLGA	Yes	n.d.	Im et al. (Ref. 118)
Femur	Rabbit^a^	Rabbit	Fibrin	Yes	>8 weeks	Dragoo et al. (Ref. 117)
OA	Rabbit^a^	Rabbit	(i.a. injection)	No	Nonlesional regions	Desando et al. (Ref. 115)
OA	Rabbit	Rabbit	(i.a. injection)	No	n.d.	Toghraie et al. (Ref. 116)
OA	Mouse	Mouse	(i.a. injection)	No	Nonlesional regions	ter Huurne et al. (Ref. 114)

^a^Autologous setting.

Abbreviations: i.a., intraarticular; n.d., not determined; OA, osteoarthritis model; PGA, polyglycolic acid; PLA, polylactic acid; PLGA, polylactic acid/polyglycolic acid co-polymer; β-TCP, β-tricalcium phosphate.

Besides artificial OA induction, the capacity of ASC to repair surgical cartilage incisions has been investigated (Table 3). In a scheme similar to ACT, ASC were first harvested, expanded and subjected to chondrogenic pre-induction for 2–3 weeks in vitro. Cells were then loaded on diverse scaffolds and re-implanted into the lesion site. Corresponding studies were conducted in rabbit (Refs 117, 118) and pig (Ref. 119) and differ in pre-induction methods and scaffold composition, but improved healing was consistently reported compared to cell-free matrix baseline conditions. Among these studies, only Dragoo et al. analysed the persistence of donor cells, making use of ASC expressing a LacZ reporter gene (Ref. 117). All explants showed ASC remaining 8 weeks after transplantation, but their direct contribution to cartilage tissue was not investigated. Notably, approaches for cartilage regeneration without in vitro pre-induction of ASC have so far not been described, although BMSC were successfully used in such a setting (Refs 120, 121, 122). Thus, the question of functional equivalence of BMSC and ASC in cartilage repair studies cannot be judged from the current literature, and a direct comparison of both cell sources under identical conditions is highly desired. Importantly, a lack of osteochondral commitment of expanded ASC, although negating their skeletal stem cell activity, may not be a major disadvantage if high trophic activity is paramount for therapeutic action for tissue regeneration and can be achieved with this cell type.

### Site-dependant bone repair capacity of ASC

The majority of ASC-based tissue engineering approaches are directed at orthotopic in vivo formation of bone (Table 4). Across all of these studies, a well agreed upon point is that repair of defective bone by ASC can be achieved when transplantation is preceded by extensive pre-differentiation protocols (Refs 101, 123, 124, 125, 126, 127, 128) or genetic manipulation with genes encoding for bone inducers (Refs 129, 130, 131, 132, 133). Overexpression of BMP-2 represents the most common strategy for the latter technique, using transgenic ASC for local growth factor delivery, rather than expecting spontaneous differentiation into osteoblasts. BMP-2 overexpression in ASC has also been used to substitute strategies that include immobilisation of recombinant BMP-2 protein to scaffolds prior to implantation (Refs 134, 135, 136). Although these cell-free approaches reproducibly led to good healing efficiency, combinations of ASC and recombinant BMP-2 were also used (Refs 137, 138, 139, 140, 141, 142) (not listed in Table 4), but only Levi et al. described that ASC improved defect repair in comparison to the BMP-loaded scaffold alone (Ref. 142).

**Table 4.** Summary of orthotopic bone formation studies with ASC

Pre-induction	Defect	ASC donor species	Acceptor species	Scaffold	Long-term engraftment	Author
	Cranium					
Yes	Calvaria	Rat	Rat	NanoBCP	n.d.	Lin et al. (Ref. 101)
Yes	Palate	Rat	Rat	PLA	n.d.	Conejero et al. (Ref. 123)
Yes	Parietal	Human	Rat	PCL/PLGA/TCP	n.d.	Kim et al. (Ref. 127)
Yes	Parietal	Human	Rat	PLGA	n.d.	Yoon et al. (Ref. 128)
Yes	Parietal	Dog^a^	Dog	Coral scaffold	n.d.	Cui et al. (Ref. 124)
Yes	Parietal	Rabbit^a^	Rabbit	PLA	n.d.	Di Bella et al. (Ref. 125)
Yes	Parietal^b^	Rabbit^a^	Rabbit	Gelatin foam	n.d.	Dudas et al. (Ref. 126)
	Long bone					
Yes^c^	Femur	Human	Rat	CP-collagen	n.d.	Peterson et al. (Ref. 132)
Yes^c^	Femur	Rabbit	Rabbit	PLGA	4 weeks	Lin et al. (Ref. 131)
Yes^c^	Ulna	Minipig	Minipig	ABM	n.d.	Chen et al. (Ref. 129)
Yes^c^	Radius	Rabbit^a^	Rabbit	PLA/PCL	n.d.	Han and Li (Ref. 130)
	Vertebral					
Yes^c^	Coccyx^b^	Pig	Rat	Fibrin	>12 weeks	Sheyn et al. (Ref. 133)
	Cranium					
No	Calvaria	Human, mouse, dog	Mouse	Apatite-PLGA	n.d.	Levi et al. (Ref. 145)
No	Mandible	Human	Rat	Fibrin	n.d.	Streckbein et al. (Ref. 146)
No	Mandibula^b^	Pig	Pig	(i.v., i.d.)	n.d.	Wilson et al. (Ref. 147)
No	Palate	Rat	Rat	PLA	n.d.	Conejero et al. (Ref. 123)
No	Parietal	Mouse	Mouse	Apatite-PLGA	>12 weeks	Cowan et al. (Ref. 144)
No	Parietal	Human	Mouse	Apatite-PLGA	2 weeks	Levi et al. (Ref. 142)
No	Parietal	Rabbit^a^	Rabbit	PLA	n.d.	Di Bella et al. (Ref. 125)
No	Parietal	Human	Rat	PLGA	n.d.	Yoon et al. (Ref. 128)
No	Parietal^b^	Rabbit^a^	Rabbit	Gelatin foam	n.d.	Dudas et al. (Ref. 126)
	Long bone					
No^d^	Femur	Human	Rat	CP-collagen	n.d.	Peterson et al. (Ref. 132)
No^d^	Femur	Rabbit	Rabbit	PLGA	4 weeks	Lin et al. (Ref. 131)
No^d^	Ulna	Minipig	Minipig	ABM	n.d.	Chen et al. (Ref. 129)
No^d^	Radius	Rabbit^a^	Rabbit	PLA/PCL	n.d.	Han and Li (Ref. 130)
No	Tibia	Sheep^a^	Sheep	Healos^©^	n.d.	Niemeyer et al. (Ref. 143)

^a^Autologous setting.

^b^Noncritical size.

^c^Transgene expression.

^d^Control group of the study.

Abbreviations: ABM, acellular bone matrix; BCP, biphasic calcium phosphate; CP, calcium phosphate ceramic; i.a., intraarticular; i.d., intra defect; n.d., not determined; PCL, polycaprolactone; PLA, polylactic acid; PLGA, polylactic acid/polyglycolic acid co-polymer; β-TCP, β-tricalcium phosphate.

Controversial data exist regarding the performance of expanded but otherwise untreated ASC in the context of long bone repair, although only a limited number of studies is available (Table 4). When nontransgenic or mock-transduced ASC were loaded on carrier matrices and transplanted into long-bone defects, no bone formation was observed (Refs 129, 131, 132). In a sheep long-bone model, ASC were unable to induce defect bridging, while BMSC facilitated defect regeneration in the same setting (Ref. 143). A closer look at the ASC control groups of the above studies further confirms the impression that pre-differentiation or genetic manipulation of ASC is a prerequisite for stimulation of bone formation. This applies even for orthotopic sites in long bones*,* where the microenvironment is rich in osteoinductive proteins which are released from the defect endings. To our knowledge, the only exception when nontransduced ASC led to substantial bone formation in the context of long-bone repair is a study by Han and Li, in which ASC were used as a control to Runx2-overexpressing cells. Possibly, the surgical connection of the implant to the vascular network was the key to the positive results of this study (Ref. 130).

Dissimilar to long-bone repair, healing of critical size defects in the cranium appears to be less challenging with untreated ASC, since bone formation without any in vitro pre-differentiation was reported in at least four studies (Refs 144, 145, 146, 147). Furthermore, untreated ASC that were transplanted as controls for newly established repair strategies also generated considerable amounts of bone (Refs 125, 128, 142), although complete absence of defect repair by ASC control groups has also been described (Refs 123, 126). Thus, orthotopic bone formation by uninduced ASC appears to be site-dependent and favoured by characteristics of the cranial microenvironment that are not present in long bones. Origin from the ectodermal germ layer, development via the intramembranous pathway and enhanced blood supply differentiates bone in the cranium from long bones. Thus, it is tempting to speculate that one major advantage in the cranium may be the denser vascular network of skull bones, which is especially interesting in the context that, beyond an absent osteochondral commitment, a lower angiogenic signature was noted for ASC (Refs 42, 59, 66, 148) and orthotopic bone formation by ACS can be triggered by co-transplantation of endothelial cells (Ref. 127).

If the trophic activity of ASC is the most crucial for stimulation of bone repair, a lower requirement for attraction and stimulation of endothelial progenitors could explain the better performance of ASC in the cranium. In line with this, a persistence of donor ASC could not be detected for more than 2 to 4 weeks after transplantation, even in settings where complete cranial defect repair was observed (Refs 131, 142). In sharp contrast, a single study by Cowan et al. reported that transplantation of uninduced ASC led to stable engraftment of the cells in a cranial defect and over 95% of nuclei in the newly formed bone were donor-derived after 12 weeks (Ref. 144). As it is not apparent which specific experimental parameters have enabled this exceptional engraftment, analogous success is waiting for repetition. Overall, particular success of ASC in cranial but not long-bone defects suggests that, in view of their low osteochondral and angiogenic signature, ASC affect bone regeneration most probably via their trophic activity than by in situ differentiation to osteoblasts with long-term persistence. Additional precise studies must unravel the contribution of host and donor cells to tissue repair as well as the influence of scaffolds, pre-cultivation, species and defect site in order to reach consensus on the main mechanisms driving ASC-dependent promotion of osteoarticular repair despite lower osteogenic and angiogenic signatures and an apparent lack of skeletal stem cell properties.

## Conclusion

**Figure 1.**
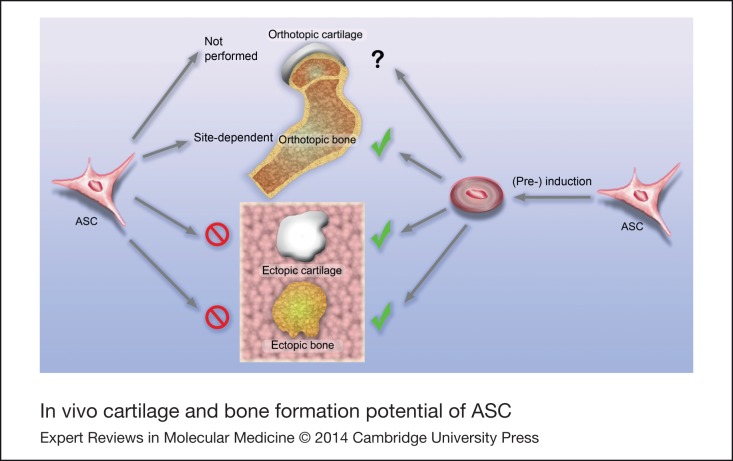
**In vivo cartilage and bone formation potential of ASC**: site dependence and requirement for pre-differentiation.

More than 50 in vivo studies have been performed to date in order to verify the potential of ASC to be used for osteoarticular regeneration. In each of the quite heterogeneous experimental setups, specific protocols were established that either enabled chondrogenic or osteogenic differentiation of the cells or that resulted in positive effects on defect healing. Regarding the greater accessibility of ASC compared to BMSC, these data are entirely encouraging for the future use of ACS in skeletal regenerative medicine. However, it is now clear that ASC do not exhibit the same degree of osteoarticular predetermination as BMSC and more manipulation is required to drive ASC into the chondrogenic or osteogenic lineage (Fig. 1). The observations that the spontaneous formation of an ectopic bone organ by BMSC cannot be reproduced with ASC and that orthotopic bone formation is only stimulated at favoured sites confirm this issue and thereby exclude a skeletal stem cell identity for ASC. Altogether, a review of the literature suggests that mainly trophic functions determine the therapeutic outcome after ASC application. Future research is needed on a direct comparison of BMSC and ASC in osteoarticular therapy to decide where and how successful BMSC protocols have to be modified to achieve promising results with ASC.
